# Expression and Functionality Study of 9 Toll-Like Receptors in 33 Drug-Naïve Non-Affective First Episode Psychosis Individuals: A 3-Month Study

**DOI:** 10.3390/ijms21176106

**Published:** 2020-08-25

**Authors:** Maria Juncal-Ruiz, Laura Riesco-Davila, Javier Vazquez-Bourgon, Victor Ortiz-Garcia de la Foz, Jacqueline Mayoral-Van Son, Rosa Ayesa-Arriola, Esther Setien-Suero, Juan Carlos Leza, Marcos Lopez-Hoyos, Benedicto Crespo-Facorro

**Affiliations:** 1 Department of Psychiatry, Hospital Sierrallana, IDIVAL, CIBERSAM, School of Medicine, University of Cantabria, 39300 Torrelavega, Spain; 2 Department of Immunology, Hospital Universitario A Coruña, 15006 A Coruña, Spain; Laura.Riesco.Davila@sergas.es; 3 Department of Psychiatry, Hospital Universitario Marqués de Valdecilla, IDIVAL, CIBERSAM, School of Medicine, University of Cantabria, 39008 Santander, Spain; javier.vazquez@scsalud.es (J.V.-B.); newvtro@gmail.com (V.O.-G.d.l.F.); rayesa@idival.org (R.A.-A.); esetien@idival.org (E.S.-S.); 4 Department of Psychiatry, Hospital Universitario Virgen del Rocío, Instituto de Investigacion Sanitaria de Sevilla (IBiS), CIBERSAM, School of Medicine, University of Sevilla, 41013 Sevilla, Spain; jacquelinem.mayoral.sspa@juntadeandalucia.es; 5 Department of Pharmacology & Toxicology, Faculty of Medicine, Complutense University (UCM), Instituto de Investigación Sanitaria (IIS) Hospital 12 de Octubre (i+12), and IUIN-UCM, CIBERSAM, 28040 Madrid, Spain; jcleza@med.ucm.es; 6 Department of Immunology, Hospital Universitario Marqués de Valdecilla, PI of “Transplant and Autoimmunity” Research Group, REDINREN RD16/0009/0027, IDIVAL, School of Medicine, University of Cantabria, 39008 Santander, Spain; marcos.lopez@scsalud.es

**Keywords:** psychosis, neuroinflammation, toll-like receptors, cytokines

## Abstract

Toll-like receptors (TLRs) are a pivotal component of the innate immune system that seem to have a role in the pathogenesis of psychosis. The purpose of this work was to compare the expression and functionality of 9 TLRs in three peripheral blood mononuclear cells (PBMCs) (monocytes, B cells, and T cells) between 33 drug-naïve first-episode psychosis (FEP) individuals and 26 healthy volunteers, at baseline and after 3-month of antipsychotic treatment. The expression of TLRs 1–9 were assessed by flow cytometry. For the assessment of the TLR functionality, cells collected in sodium heparin tubes were polyclonally stimulated for 18 h, with different agonists for human TLR1–9. The results of our study highlight the role that TLR5 and TLR8 might play in the pathophysiology of psychosis. We found a lower expression of these receptors in FEP individuals, regarding healthy volunteers at baseline and after 3-month of treatment on the three PBMCs subsets. Most TLRs showed a lower functionality (especially reduced intracellular levels of TNF-α) in patients than in healthy volunteers. These results, together with previous evidence, suggest that individuals with psychosis might show a pattern of TLR expression that differs from that of healthy volunteers, which could vary according to the intensity of immune/inflammatory response.

## 1. Introduction

The role of infection and immunity in psychosis etiology was investigated for more than a century [1]. Exposure to infectious agents or inflammatory response during pregnancy might be associated with a disruption of fetal development and is proposed as a neurodevelopmental model of schizophrenia [2,3].

Toll-like receptors (TLRs) are a pivotal component of the innate immune system that are expressed by various subsets of immune and non-immune cell types [4,5], including central nervous system (CNS) cell types, such as microglia, astrocytes, and neurons [6]. These inflammatory cells play a crucial role in the generation of neuroinflammation involved in the immunopathogenesis of different CNS diseases [7]. In this line, several original studies [8,9,10] and meta-analysis [11,12] described an increase of proinflammatory cytokines and chemokines in first episode psychosis (FEP) individuals, which normalize after antipsychotic treatment. 

The production of proinflammatory cytokines and free radicals of oxygen and nitrogen, as well as the sustained activation of microglia during the first stages of life, and especially following maternal infection and inflammation, would lead to a reduction in glutamatergic neurons in some brain areas [13,14,15,16], similar to the glutamatergic hypofunction found in patients with schizophrenia [17]. Impaired immune response, along with biological, genetic, and environmental factors, might in turn, lead to psychotic disorders [18].

It appears that TLR level clearly influences the intensity of immune/inflammatory responses, indicating that decreased TLR expression protects against excessive cell stimulation via exogenous or endogenous ligands, and is a counterbalancing mechanism that limits the excessive development of inflammation [19,20].

Up to now, there are few publications that have studied TLR expression or functionality in psychosis, most of them on chronic schizophrenia individuals [19,21,22,23,24] but one in FEP [25]. It is worth mentioning that there are several methodological caveats in previous research that preclude the drawing of any solid conclusion. In all previous studies [19,21,22,23,24], except Kéri et al. [25], subjects were on medication, and several studies suggest that antipsychotics might influence TLR expression [25,26]. In addition, most of these studies were not controlled for some of the most important confounding variables, such as body mass index (BMI) [23], as well as cannabis consumption [19,21,22,23,25], and smoking [21,23] behavior. Further, no previous publication took into account the use of concomitant medication that might also alter the immune response [27]. Finally, it is important to consider that samples from previous studies that focus on patients with chronic schizophrenia were often heterogeneous, and consisted of subjects with significant differences in the predominant clinical symptoms or stage of the disease that could differ in terms of inflammatory and immunological alterations [19,20].

The aims of the present study were (i) to investigate the expression and functionality of 9 TLRs in three different peripheral blood mononuclear cells (PBMCs), monocytes, B cells and T cells, in FEP individuals and healthy volunteers, at baseline and after 3-month of antipsychotic treatment in the patient group; and (ii) to examine both the patterns of TLR expression and functional changes after 3 months of antipsychotic treatment within the FEP individuals group.

Our hypothesis is that we will find differences both in the expression pattern of TLRs and in their functionality between subjects with FEP and healthy volunteers. After 3-month of antipsychotic treatment, we will see changes in the expression and functionality of TLRs in patients. Considering that the level of expression of TLRs and the intensity of the immune/inflammatory response influence each other [20], the pattern of expression of TLRs might vary throughout the evolution of the disease. We hope to find a different expression pattern of TLRs, compared to other studies based on chronic schizophrenia.

## 2. Results

### 2.1. Differences in Sociodemographic and Clinical Variables between Groups

As shown in Table 1, no statistically significant differences were found in the demographic and clinical variables between healthy volunteers and FEP individuals, at baseline (*p* values > 0.1). Out of 33 patients, 19 (57.6%) were initially assigned to the aripiprazole group, whereas 14 (42.4%) were assigned to the risperidone group; this was a non-statistically significant random distribution (*z* = 0.87; *p* = 0.384).

Within-subject analysis showed that BMI increased significantly, following 3-month of antipsychotic treatment (*t* = 3.71; *p* = 0.002), although no differences were found regarding BMI between healthy volunteers and FEP individuals at 3-month. We observed a general improvement in the total scores of clinical scales after 3-month of treatment (*p* values < 0.01) except in the SANS (*U* = 0.86; *p* = 0.376).

### 2.2. Comparison of TLR Expression Healthy Volunteers and Patients

**Figure 1:**
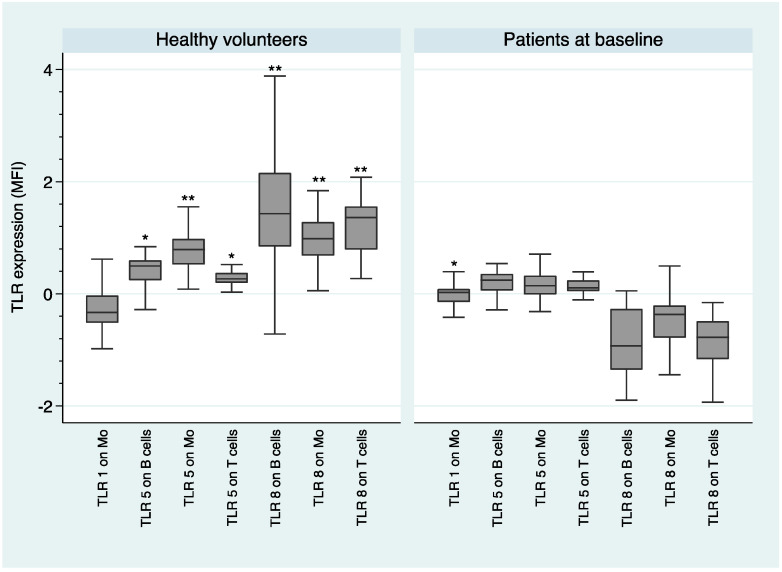
Comparison of toll-like receptors (TLR) expression between healthy volunteers and patients at baseline. log-transformed data. * *p* < 0.01; ** *p* < 0.001; Mo—Monocytes; MFI—mean fluorescence intensity.

Regarding TLR expression between healthy volunteers and patients at baseline, Figure 1 shows that the expression of TLR1 on monocytes was higher in the patient group with respect to healthy volunteers (*U* = 2.87; *p* = 0.004). However, healthy volunteers had a higher expression of TLR5 and TLR8 than the FEP individuals on the three PBMCs subsets—TLR5 on B cells (*U* = −2.64; *p* = 0.008), monocytes (*U* = −3.60; *p* = 0.000), and T cells (*U* = −2.85; *p* = 0.004); TLR8 on B cells (*U* = −5.85; *p* = 0.000), monocytes (*U* = −6.20; *p* = 0.000), and T cells (*U* = −6.2; *p* = 0.000).

**Figure 2:**
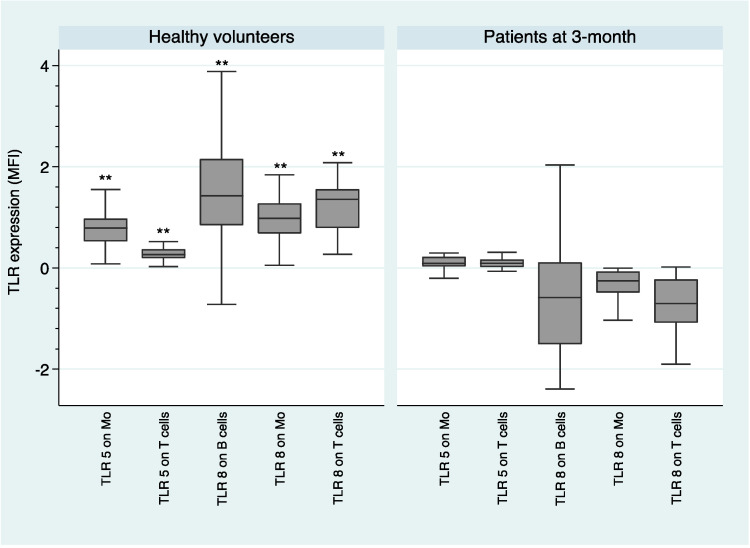
Comparison of TLR expression between healthy volunteers and patients at 3-month. log-transformed data. **: *p* < 0.001; Mo: Monocytes; MFI—mean fluorescence intensity.

Figure 2 shows that healthy volunteers had a higher expression of TLR5 on monocytes (*U* = −4.0; *p* = 0.000) and T cells (*U* = −3.66; *p* = 0.000), as well as a higher expression of TLR8 on B cells (*U* = −3.18; *p* = 0.001), monocytes (*U* = −4.22; *p* = 0.000), and T cells (*U*= −4.15; *p* = 0.000) than FEP individuals, after 3-month of antipsychotic treatment.

**Figure 3:**
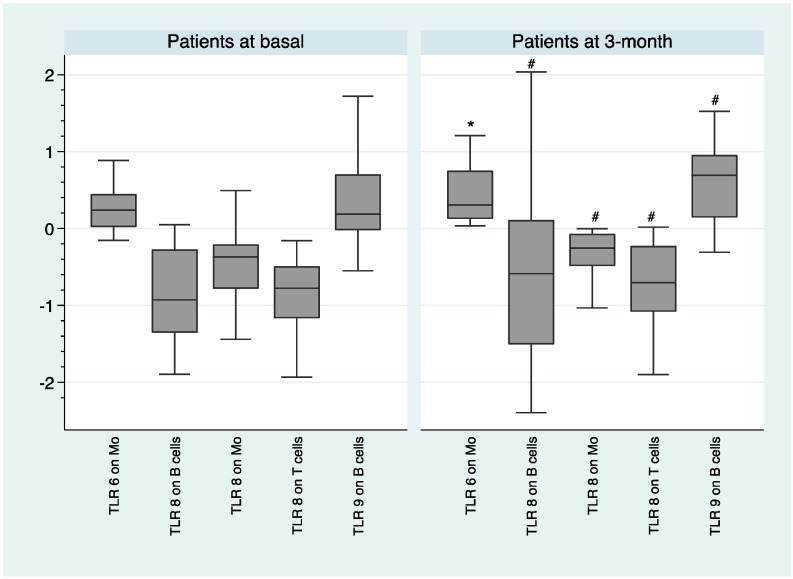
Within-subject analysis of the expression of TLRs after 3-month of antipsychotic treatment. log-transformed data. *: *p* < 0.01; #: trend toward statistical significance; Mo—monocytes; MFI—mean fluorescence intensity.

Within-subject analysis shows that after 3-month of antipsychotic treatment FEP individuals had statistically significant higher expression level of TLR6 on monocytes (*F* = 12.77; *p* = 0.003) and we observed a trend toward a significant higher expression levels of TLR8 on monocytes (*F* = 6.61; *p* = 0.022), B cells (*F* = 7.53; *p* = 0.023), and T cells (*F* = 6.09; *p* = 0.027), as well as TLR9 on B cells (*F* = 8.23; *p* = 0.014), as shown in Figure 3.

### 2.3. Comparison of IL-1β, IL-6, and TNF-α Intracellular Concentrations Following TLR Stimulation between Healthy Volunteers and Patients 

**Figure 4:**
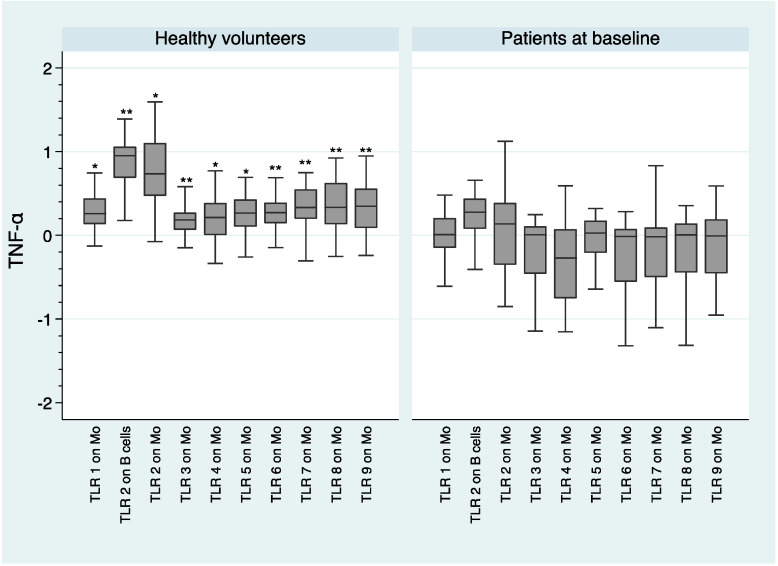
Comparison of intracellular production of TNF-α following TLR stimulation between healthy volunteers and patients at baseline. log-transformed data. *: *p* < 0.01; **: *p* < 0.001; Mo—monocytes.

Figure 4 shows significantly higher intracellular levels of TNF-α in healthy volunteers compared to patients after baseline stimulation of the following TLRs on monocytes: TLR1 (*U* = −2.79; *p* = 0.005), TLR2 (*U* = −3.08; *p* = 0.002), TLR3 (*U* = −3.41; *p* = 0.001), TLR4 (*U* = −2.90; *p* = 0.004), TLR5 (*U* = −2.79; *p* = 0.005), TLR6 (*U* = −4.03; *p* = 0.000), TLR7 (*U* = −4.31; *p* = 0.000), TLR8 (*U* = −3.57; *p* = 0.000), and TLR9 (*U* = −3.34; *p* = 0.001). In addition, significantly higher levels of TNF-α were found in healthy volunteers following TLR2 stimulation on B cells (*U* = −3.60; *p* = 0.000). No differences were found in the intracellular production of IL-1β, neither in IL-6 between both groups at baseline (*p* values > 0.1).

**Figure 5:**
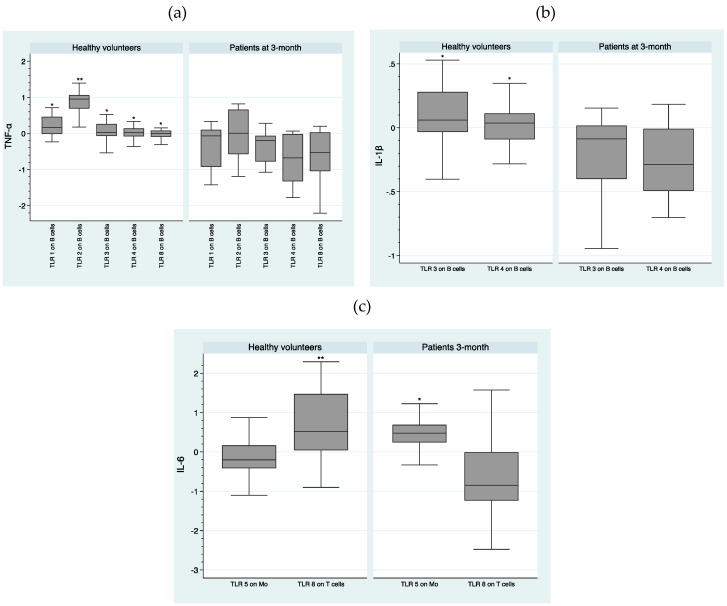
Comparison of intracellular production of (**a**) TNF-α, (**b**) IL-1β, and (**c**) IL-6, following TLR stimulation between healthy volunteers and patients at 3-month. log-transformed data. *: *p* < 0.01; **: *p* < 0.001. Mo—monocytes.

After the FEP individuals were under antipsychotic treatment for 3-month, we found higher intracellular levels of the following cytokines in healthy volunteers as compared to patients (Figure 5a)—significant higher expression of TNF-α after TLR1 stimulation on B cells (*U* = −2.92; *p* = 0.003), TLR2 stimulation on B cells (*U* = −3.77; *p* = 0.000), TLR3 stimulation on B cells (*U* = −2.85; *p* = 0.004), TLR4 stimulation on B cells (*U* = −3.17; *p* = 0.002), and TLR8 stimulation on B cells (*U* = −2.61; *p* = 0.009). Figure 5b shows a significantly higher production of IL-1β, following TLR3 stimulation on B cells (*U* = −2.57; *p* = 0.010) and TLR4 stimulation on B cells (*U* = −2.76; *p* = 0.006) in healthy volunteers. Figure 5c shows significantly higher intracellular levels of IL-6, following TLR8 stimulation on T cells (*U* = −3.18; *p* = 0.001) in healthy volunteers and a significantly higher intracellular production of IL-6 in the FEP individuals group, after TLR5 stimulation on monocytes (*U* = 2.61; *p* = 0.009).

Within-subject analysis did not find statistically significant differences in the intracellular cytokine production after 3-month of antipsychotic treatment (*p* values > 0.1).

## 3. Discussion

The results of our study showed a lower expression of TLR5 and TLR8 in FEP individuals as compared to healthy volunteers at baseline and after 3-month of treatment on the three PBMCs subsets. Most TLRs showed a lower functionality (especially reduced intracellular levels of TNF-α) in patients than in healthy volunteers, mainly on the monocytes at baseline and on B cells after medication. Both cell types, but not T cells, are antigen-presenting cells in which the altered expression and function of TLRs could have impacted in the inflammatory response of FEP. Within-subject analysis showed a tendency of an up-regulation of the expression of some TLRs after 3-month of antipsychotic treatment.

Considering our findings, we focused on the possible pathophysiological implications of impaired expression and functionality of TLR5 and TLR8. TLR5 is expressed in lung and intestinal epithelial cells, human endometrium, bladder, granulosa cells leukocytes, adipocytes, and some cancer cells, among others. Recent research studies described that the correct expression of TLR5 during neonatal period influences the long-term gut microbiota composition [28], and the alteration of microbiota was related to psychosis in at least a subgroup of patients [29,30,31]. Moreover, the alteration of the TLR5 expression was described in individuals with insulin resistance, which is a very prevalent condition in subjects with schizophrenia [32,33]. It was also recently related to schizophrenia polygenic risk score and a good clinical response to antipsychotic drugs [34].

TLR8, an endosomal sensor of RNA degradation products in human phagocytes, is involved in the recognition of viral and bacterial pathogens through the modulation of the suppressive activity of regulatory T cells [35]. Several investigations showed that murine TLR8 plays a pivotal role in the regulation of myeloid cells and prevention of autoimmunity, by controlling TLR7 expression [36,37]. In this sense, in this study we also found an altered functionality response in B cells, after 3 months of treatment. Altered B cell signaling might also increase the risk of autoimmunity [38], and as is known, autoimmunity is related to the etiology of schizophrenia [39,40].

Thus far, only a handful of studies evaluated the expression and functionality of TLRs in schizophrenic individuals. Chang et al. [23] described a lower mRNA expression of TLR5 and TLR3, as well as an elevated mRNA expression of IL-6 and IL-10 in monocytes, in patients with chronic schizophrenia as compared to healthy volunteers. A recent study by Kozłowska et al. [19] found that mRNA expression of TLR1, TLR2, TLR4, TLR6, and TLR9 were lower in chronic schizophrenic individuals, TLR3 and TLR7 manifested a higher mRNA expression in chronic schizophrenia, while TLR5 and TLR8 mRNAs did not differ between patients and healthy volunteers. In the unique study on FEP, Kéri et al. [25] indicated that treatment-naïve psychosis individuals displayed an increased expression of TLR4 on monocytes, as well as TLR5 on monocytes and T cells. The initial high expression of TLR4 on monocytes was normalized after 2-month of treatment and an up-regulation of TLR2 on both monocytes and T cells was also observed. 

Some of the differences between these studies with each other and with respect to our findings could be explained if we consider the methodological limitations described in the introduction section. In addition, we suggest that the immune response probably could not be defined categorically (elevated or decreased), but should be understood as a spectrum in which the expression and functionality of TLRs could vary, depending on various factors, such as the clinical situation (compensation vs. relapse), the presence of an infection or autoimmunity, the intensity of the inflammatory response, the time under antipsychotic treatment, etc. We speculated that decreased TLR expression and functionality might be a consequence of an excessive cell stimulation via exogenous or endogenous ligands, through a counterbalancing mechanism limiting the excessive development of inflammation [19,20]. Within-subject analysis, in which an up-regulation of the expression of some TLRs, such as TLR8, was observed after 3 months of antipsychotic treatment, further support the counterbalancing mechanism hypothesis (see Figure 3). The fact that the expression of those TLR that were up-regulated in the within-subject analysis at 3-month, continued to be lower than those of healthy volunteers, suggests that more time under treatment was probably needed for the counterbalancing mechanism to be corrected. 

This hypothesis would help to understand why several studies found a different pattern of TLR expression, as their samples could be constituted by individuals in different stages of the disease and under different antipsychotic treatments, in which the immune response could thus vary, considering all clinical and immunological variables described above [19]. Therefore, it seemed that all previous studies coincided in observing an alteration in the expression and functionality of some TLRs in schizophrenia subjects, and their findings could be different, depending on the degree of activation of the immune system, which in turn was affected by other clinical and immunological variables. Considering our findings, we proposed to study in more detail how the expression and functionality of TLR5 and TLR8 vary longitudinally, in order to understand the possible relationship between clinical evolution and the pattern of expression of some TLRs. A better understanding of how this pattern changed over time could help us to develop biomarkers that help in the follow-up of these patients and to develop new therapeutic targets.

The fact that we observed an alteration in the innate immune response (monocytes) and in the adaptive immune response (B cells and T cells), both at the onset of the disease and after 3 months of antipsychotic treatment (see Figure 1, Figure 2, Figure 3, Figure 4 and Figure 5), suggests that both types of response could play an important role in the dysregulation of the immune system that we observed in subjects with schizophrenia. Antipsychotic treatment seemed to have a regulatory effect in both immune responses (see Figure 3). However, taking into account that this was the first study in which the expression and functionality of 9 TLRs in three different cell types were analyzed, we must be cautious with the results obtained and especially with the formulation of possible hypotheses. Further studies are needed to better explore the possible connection link between TLRs and different immune cell types.

Our findings, together with previous evidence, might be consistent with the neurodevelopmental model of schizophrenia, in which a prenatal infection or inflammation might produce a change in both brain areas and immune system, which increase the future risk to develop both psychosis disorders and immune disturbances, like autoimmunity [40,41]. It is described as a sustained activation of the immune system that produces a long-term sustained activation of the microglia, which was linked to neurotoxic processes and an increased risk of developing some mental illnesses like psychosis [16,42]. However, the origin of this inflammation could vary, depending on each individual. Each TLR is activated by different substrates and pathways, and this might explain why different expression patterns are found in different samples of psychosis individuals. This also supports the idea of “personalized medicine”. Considering that the immune response is affected by different variables and factors that can vary in each person, the main objective is probably not to find a single common alteration in any TLR in all individuals with schizophrenia but to observe what initial alterations exist in the pattern of TLR expression in each subject at the beginning of the first episode of psychosis, and to carry out a longitudinal follow-up over time. Analyzing how this pattern varies over time, along with other clinical evaluations could help us make therapeutic decisions.

This study has several limitations that should be considered—first, stress level, which is known to influence inflammatory status, was not measured. Second, we did not consider the consumption of classical analgesic or anti-inflammatory drugs as possible confounding factors. However, it is important to remark that the subjects in this study were young, healthy individuals without chronic diseases or chronic treatments. Third, analysis was performed on peripheral blood. We assumed that these findings were an indicator of what occurred in the CNS and, therefore, we would expect to find a greater disturbance of TLR expression and intracellular cytokine production in the CNS.

## 4. Materials and Methods 

### 4.1. Study Setting

The data for the present study were obtained from a large epidemiological cohort of patients who were treated in a longitudinal intervention program of FEP (PAFIP: Programa de Atención a Fases Iniciales de Psicosis) conducted at the University Hospital Marqués de Valdecilla in Cantabria, Spain. The main procedures that were carried out in this program are described elsewhere [43]. The study was approved by the Cantabria Ethics Institutional Review Board (Comité Ético de Investigación Clínica de Cantabria), conforming to international standards for research ethics (Approval Number: 2016.119, 22 July 2016). Patients meeting inclusion criteria and their families provided written informed consent to be included in the PAFIP. The randomized trial approval number: ClinicalTrials.gov ID: NCT02897167 (13/09/2016). “Study of the Activation of Proinflammatory Pathways of Toll-like Receptors in Schizophrenia Patients (P AFIP_TLR)”. https://clinicaltrials.gov/ct2/show/NCT02897167.

### 4.2. Subjects

From October 2016 to March 2019, 33 individuals meeting the following inclusion criteria—age between 15 and 50; residency in the catchment area; experiencing their first episode of psychosis; no prior treatment with antipsychotic medication (not even a single dose of antipsychotic medication before blood test), and DSM-IV criteria for brief psychotic disorder, schizophreniform disorder, schizophrenia, schizoaffective disorder, or psychotic disorder not otherwise specified, were selected as entry in the present investigation. Patients were excluded if they met any of the following criteria—DSM-IV criteria for drug dependence; DSM-IV criteria for mental retardation; history of neurological disorder or brain injury; history of inflammatory diseases or immunodeficiency, and recent (1 week) use of glucocorticoids or vaccination less than 3 months ago. The diagnoses were confirmed by an experienced psychiatrist, applying the Structured Clinical Interview for DSM-IV (SCID-I), 6 months after the baseline visit.

In the same period of time, we also selected 26 healthy volunteers with similar characteristics to patients, according to the following variables—sex, age, body mass index (BMI), and cannabis and tobacco consumption. Healthy volunteers were excluded if they had—DSM-IV criteria for drug dependence; DSM-IV criteria for mental retardation; first-degree relatives with a diagnosis of psychosis; history of neurological disorder or brain injury; history of inflammatory diseases or immunodeficiency, and recent (1-week) use of glucocorticoids or vaccination less than 3-month ago.

### 4.3. Study Design

This was a prospective, randomized, flexible dose, open-label study. At baseline blood sampling, all patients were antipsychotic naïve. A computer generated a randomization list based on a simple randomization procedure to select at baseline the antipsychotic used between aripiprazole or risperidone. At the treating physician’s discretion, the dose and type of antipsychotic medication could be changed, based on clinical efficacy and the profile of side effects during the follow-up period. Concomitant medication, such as antimuscarinics, benzodiazepines, antidepressants, and mood stabilizers, was considered as possible confounding factors in the analyses.

Fasting venous blood samples were collected at baseline and three months following the starting of treatment. In addition, height, weight, and BMI were measured at baseline and 3-month after medication.

We compared the expression of 9 TLRs and their functionality (measured as intracellular production of IL-1β, IL-6, and TNF-α, following TLR stimulation) between healthy volunteers and patients at baseline and at 3-month. Likewise, we performed within-subject analyses to study the threshold change of TLR expression and function after 3 months of antipsychotic treatment.

To evaluate the antipsychotic efficacy for reducing psychotic symptoms we compared the total scores of the following clinical scales at baseline and following 3-month of antipsychotic treatment—Scale for the Assessment of Positive Symptoms (SAPS), the Scale for the Assessment of Negative Symptoms (SANS), the Brief Psychiatric Rating Scale (BPRS), and the Clinical Global Impression (CGI).

### 4.4. Assessments

#### 4.4.1. Expression of TLRs in PBMCs

The cell-surface expression of TLR1, TLR2, TLR4, TLR5, and TLR6 and the intracellular expression of TLR3, TLR7, TLR8, and TLR9 were assessed on distinct PBMCs subpopulations (T cells, B cells, and monocytes) using flow cytometry, as previously described [44]. Cells collected into the sodium heparin tubes were incubated with FITC- or PE-conjugated anti-human CD19, PercP-conjugated anti-human CD3, and allophycocyanin-conjugated anti-human CD14 to identify B cells, T cells, and monocytes, respectively, and with FITC- or PE-conjugated anti-human TLR (TLR1, TLR2, TLR4; eBioscience, San Diego, CA, USA) or FITC or PE mouse IgG2a isotype control. Staining for TLR3 and TLR5 to TLR9 expression (Acris Anti-bodies, Germany; and eBioscience, respectively) was performed by permeabilizing with FACS permeabilizing solution (BD Biosciences), and staining with fluorochrome-conjugated anti-human TLR or mouse IgG2a isotype control. Expression of TLRs was gated and analyzed by flow cytometry (FACSCalibur, BD Biosciences), as the mean fluorescence intensity (MFI), using the CellQuest Pro software. Data were presented as relative MFI (MFI of TLR/MFI of isotype control).

#### 4.4.2. Assessment of TLR Functionality in Circulation PBMCs

For this purpose, we decided to quantify the intracellular expression of three of the most important proinflammatory cytokines (IL-1β, IL-6, and TNF-α), after TLR stimulation. Cells collected in sodium heparin tubes were polyclonally stimulated for 18 h, with different agonists for human TLR1–9 (InvivoGen, San Diego, CA, USA), in the presence or absence of Brefeldine A (Sigma-Aldrich, St. Louis, MO, USA) in polypropylene tubes. As a negative control, cells were incubated in identical medium without stimulus. After culture, cells were permeabilized with FACS permeabilizing solution (BD Biosciences) and intracellularly stained with PE-labeled mAb against cytokines (IL-1β, IL-6, TNF-α; BD Biosciences) and analyzed by flow cytometry. Percentage of intracellular cytokine-producing B cells, T cells, and monocytes was analyzed using the CellQuest Pro software (BD Biosciences), as previously described [44].

### 4.5. Statistical Analysis 

The Kolmogorov–Smirnov test was used to examine the normality of data. Demographics and clinical measures were analyzed using T-Student test, W-Wilcoxon rank-sum test, or Chi-Square test as necessary. T-Student test for dependent data and W-Wilcoxon signed-rank test were used to assess the change of BMI and clinical scales within the patient group after 3-month of treatment. 

Wilcoxon rank-sum test for independent data was used to compare the expression and functionality of TLRs between healthy volunteers and patients at baseline and after antipsychotic treatment. 

Repeated measures Analysis of Covariance (ANCOVA) with BMI as covariate was used in the within-subject analysis. BMI was used as covariate as statistically significant changes were found with regards to this variable during the follow-up period within the sample of FEP individuals. In addition, previous studies described the influence of BMI on cytokine levels [45].

Median MFI expression of TLRs and median intracellular levels of cytokines and interquartile ranges were represented by Box Plots after performing a natural logarithmic transformation. Only the statistically significant differences are shown in the figures, because otherwise it would take many figures to show clearly all the data concerning the expression and functionality of the 9 TLRs. However, all the expression and functionality data of the 9 TLRs are available upon request.

As many tests were conducted, we assumed that the probability of a Type I error (false positive) was increased. Keeping this in mind and considering our research was exploratory, we decided to use a more conservative statistical significance threshold of 0.01, instead of applying multiple-testing correction in order to avoid Type II error (false negative).

STATA 16.0 was used for statistical analysis. Statistical tests were two-tailed with a 99% confidence interval.

## 5. Conclusions

Drug-naïve patients with schizophrenia spectrum disorders exhibit a pattern of TLR expression that differs from that of healthy volunteers, and which could vary according to the intensity of immune/inflammatory response. We highlight the role that TLR5 and TLR8 might have in the pathophysiology of psychosis.

## Figures and Tables

**Table 1:** Demographic and clinical characteristics between healthy volunteers and patients

	Entire Simple (*n* = 59), *n* (%)	Healthy Volunteers Group (*n* = 26), *n* (%)	Patients Group, (*n* = 33), *n* (%)	Statistics
Sex (Male)	37 (62.7)	19 (73.1)	18 (54.6)	*Χ^2^* = 2.14; *p* = 0.144 ^(1)^
Cannabis Users	27 (46.6)	12 (46.2)	15 (46.9)	*Χ^2^* = 0.00; *p* = 0.956 ^(1)^
Tobacco Users	30 (53.6)	15 (57.7)	15 (50)	*Χ^2^* = 033; *p* = 0.565 ^(1)^
Baseline Treatment (Aripiprazole)			19 (57.6)	*z* = 0.87; *p* = 0.384 ^(3)^
	**Mean (SD)**	**Mean (SD)**	**Mean (SD)**	**Statistics**
Age at Onset (Years) *	29.9 (7.86)	29.6 (6.30)	30.2 (8.99)	*t* = 0.25; *p* = 0.803 ^(1)^
BMI (kg/m^2^)	23.7 (3.94)	24.6 (4.16)	23 (3.69) ^(a)^	*t* = −1.52; *p* = 0.135 ^(1)^
			25.3 (3.94) ^(b)^	*t* = 3.71; *p* = 0.002 ^(2)^
Total BPRS score			75.3 (19.0) ^(a)^	*U* = −3.52; *p* = 0.000 ^(2)^
			36.9 (15.59) ^(b)^	
Total SAPS Score			17 (5.17) ^(a)^	*U* = −3.52; *p* = 0.000 ^(2)^
			1.94 (3.86) ^(b)^	
Total SANS Score			5.7 (5.42) ^(a)^	*U* = 0.89; *p* = 0.376 ^(2)^
			5.8 (6.47) ^(b)^	
CGI			6.8 (0.44) ^(a)^	*U* = −3.56; *p* = 0.000 ^(2)^
			2.6 (1.84) ^(b)^	

BPRS—Brief Psychiatric Rating Scale; CGI—Clinical Global Impression; SANS—Scale for the Assessment of Negative Symptoms; and SAPS—Scale for the Assessment of Positive Symptoms. * Healthy volunteers—enrollment; Patients group—disease. ^(a)^ At baseline; ^(b)^ At 3-month. ^(1)^ Comparison between the patient group and healthy volunteers at baseline; ^(2)^ Within-subject analysis after 3-month of treatment; and ^(3)^ Comparison with a hypothesized proportion.

## Abbreviations

BMI	Body mass index
PBMC	peripheral blood mononuclear cells
CNS	Central nervous system
FEP	First-episode psychosis
PAFIP	Programa de Atención a Fases Iniciales de Psicosis
TLR	Toll-like receptors
